# The BE (2)-M17 neuroblastoma cell line: revealing its potential as a cellular model for Parkinson’s disease

**DOI:** 10.3389/fncel.2024.1485414

**Published:** 2024-11-26

**Authors:** Angel Carvajal-Oliveros, Camila Román-Martínez, Enrique Reynaud, Eduardo Martínez-Martínez

**Affiliations:** ^1^Laboratory of Cell Communication and Extracellular Vesicles, Division of Basic Science, Instituto Nacional de Medicina Genómica, Mexico City, Mexico; ^2^Departamento de Genética del Desarrollo y Fisiología Molecular, Instituto de Biotecnología, Universidad Nacional Autónoma de México, Cuernavaca, Mexico

**Keywords:** BE (2)-M17, SH-SY5Y, Parkinson’s disease, neuroblastoma, dopamine, tyrosine hydroxylase

## Abstract

Parkinson’s disease is a pathology with a wide range of *in vivo* and *in vitro* models available. Among these, the SH-SY5Y neuroblastoma cell line is one of the most employed. This model expresses catecholaminergic markers and can differentiate and acquire various neuronal phenotypes. However, challenges persist, primarily concerning the variability of growth media, expression of dopaminergic markers, and a wide variety of differentiation protocols have been reported in the literature without direct comparison between them. This lack of standardized differentiation conditions impacts result reproducibility and it makes it very difficult to compare the results obtained from different research groups. An alternative cellular model is the neuroblastoma BE (2)-M17 which exhibits a high basal expression of numerous dopaminergic markers such as tyrosine hydroxylase (TH), vesicular monoamine transporter 2 (VMAT2), and dopamine transporter (DAT). The BE (2)-M17 cells show neuronal properties, grows rapidly in conventional media, and can easily be differentiated to increase their dopaminergic phenotype. In this review, we will thoroughly explore the properties of the BE (2)-M17 cell line and discuss its potential as an excellent model for studying Parkinson’s disease.

## 1 Introduction

The rise in life expectancy, resulting from advancements in medical treatments, and overall quality of life, has promoted the appearance in the population of previously relatively uncommon age-related conditions, such as neurodegenerative diseases (Azam et al., 2021). This circumstance poses several challenges because the number of affected people is expected to increase dramatically in the following decades (Jaul and Barron, 2017; Martinez et al., 2021). Nearly 40% of the world’s population is affected by a neurological disorder, and this percentage is projected to double by 2050 (World Federation of Neurology, 2023). Ninety percent of these cases are attributed to just ten of the most common neurological disorders, including Parkinson’s disease (PD), whose incidence is expected to increase by a factor of 1.6 between 2010 and 2030. By 2030, approximately 9 million people are expected to suffer from PD, focusing only on the most populous countries (Bach et al., 2011).

Neurodegenerative diseases primarily manifest through detrimental changes in the homeostasis of the nervous system and its neuronal function (Guo et al., 2022). These alterations lead to a variety of symptoms, ranging from motor impairments, speech difficulties, and mood disturbances to more severe outcomes like memory loss (Lamptey et al., 2022). Despite an increasing research effort to elucidate cellular mechanisms over the last two decades, the field requires the development of new cellular models to facilitate physiological, cellular and molecular experimentation that will aid in the understanding of neurodegenerative diseases (Gitler et al., 2017; Guo et al., 2022).

Parkinson’s disease is considered one of the most prevalent neurodegenerative disorders and is primarily distinguished by motor symptoms (Armstrong and Okun, 2020). This impairment is attributed to a decrease of dopamine in striatum, which is directly associated with the death of dopaminergic neurons, particularly those found in the *substantia nigra pars compacta* (Ye et al., 2022). Several factors contributing to the death of these neurons have been linked to issues such as improper protein folding and degradation, mitochondrial dysfunction, oxidative stress, and neuroinflammatory processes (Poewe et al., 2017). A wide range of *in vivo* and *in vitro* models have been developed to study the molecular processes implicated in the development and progression of PD. Rodents constitute the main *in vivo* models, which account for 85% of animal studies. Followed by primates, accounting for 10% of the models used, and lastly, other animal models like *Drosophila melanogaster* and *Caenorhabditis elegans* with the remaining 5% (Konnova and Swanberg, 2018). Each of these models offers unique advantages and disadvantages, enabling the study of PD from diverse perspectives.

Although most PD cases are sporadic, the resemblance of phenotypes resulting from genetic alterations, along with the advancement of genome manipulation tools, has facilitated the development of genetically modified animals that replicate many aspects of the PD etiology (Konnova and Swanberg, 2018). Mammalian and non-mammalian models have been utilized to create animals that overexpress proteins linked to PD, such as α-Synuclein, Synphilin-1, Parkin, LRRK2, PINK1, etc., (Carvajal-Oliveros et al., 2021; De Haas et al., 2019; Hollville et al., 2020; Pinto-Costa et al., 2023; Xiong et al., 2017). Additionally, researchers have been able to assess the impact of molecules that either induce dopaminergic neuron death (MPTP, 6-OHDA, rotenone, paraquat) (Ibarra-Gutiérrez et al., 2023; Porras et al., 2012; Simola et al., 2007; Vaccari et al., 2017) or possess neuroprotective properties (nicotine, curcumin, caffeine) (Carvajal-Oliveros et al., 2023; Patel et al., 2022; Ren and Chen, 2020). In most of these animal models, processes like those occurring in the aging of the human brain are observed. Nevertheless, a notable limitation is the inability to fully replicate the neuronal or clinical phenotypes seen in patients (Jucker, 2010).

*In vitro* models have also been extensively used because they reduce the duration and costs of research processes and minimize ethical and regulatory issues, while also offering improved reproducibility of results by ensuring that they originate from the same genetic lineage (Falkenburger et al., 2016; Slanzi et al., 2020). These models closely replicate key features involved in PD pathology such as mitochondrial dysfunction, protein aggregation and ROS accumulation (Ke et al., 2021). Many of these models are derived from human cells, bridging the gap between animal studies and clinical trials on patients, thus contributing to the searching and understanding of the mechanisms associated with potential drugs (Slanzi et al., 2020). Some of the most well-known cellular models include immortalized neuronal progenitor cell line (MN9D), rat immortalized neuronal progenitor cell line (CSM14.1), mouse neuroblastoma (N2A) pheochromocytoma cells derived from the rat adrenal medulla (PC12), Human neuroglioma cells (H4), Immortalized human embryonic kidney cells (HEK 293), Lund human mesencephalic (LUHMES), primary neuronal cell cultures, iPSCs and organoids (Falkenburger et al., 2016; Ke et al., 2021; Slanzi et al., 2020). Finally, one of the most extensively characterized and widely used cell line models for PD research is the human neuroblastoma cell line SH-SY5Y (Ioghen et al., 2023), whose advantages and disadvantages will be discussed in more detail.

## 2 SH-SY5Y cell line: ode to an old friend

SH-SY5Y is an immortalized cell line derived from human neuroblastoma. SH-SY5Y is a subline of the SK-N-SH cell line which was established in 1970 from a metastatic bone tumor of a 4-year-old female (ATCC^®^- CRL-2266). In this cell line, there are two converging cellular populations: one neuroblast-like cells adherent and a low proportion of epithelial-like cells (Lopez-Suarez et al., 2022). This cellular model has been one of the most widely used for *in vitro* research, particularly for studies of dopaminergic degeneration associated with PD (Xicoy et al., 2017). From 1989 to the middle of 2024, a search using “SH-SY5Y” on PubMed yielded over 12,530 results, while a search for “SH-SY5Y and Parkinson’s” gave approximately 2,370 results (NIH, PubMed^®^).

SH-SY5Y has significant advantages widely described, such as the expression of dopaminergic, cholinergic, and glutamatergic markers such as L-amino acid decarboxylase, tyrosine hydroxylase, dopamine beta-hydroxylase, choline acetyltransferase, acetylcholine esterase, vesicular glutamate transporter, and glutamate decarboxylase (Filograna et al., 2015). However, there are discrepancies in these findings across various publications, primarily due to the considerable diversity in growth media, and differentiation protocols (Kovalevich and Langford, 2013). This represents a significant dilemma when working with the SH-SY5Y cell line. The ATCC^®^ recommends employing Eagle’s Minimum Essential Medium and F12, supplemented with fetal bovine serum (FBS) at a final concentration of 10% (ATCC^®^). However, research studies diverge in their selection of culture medium for this cell line. Most articles cite the use of Dulbecco’s Modified Eagle’s Medium (DMEM), followed by alternatives like Minimum Essential Medium (MEM), Roswell Park Memorial Institute medium (RPMI), Cosmedium-001, among others. Alongside the variability in culture media, there are a wide range of fetal bovine serum supplementation, varying from 5 to 20% (Xicoy et al., 2017).

SH-SY5Y is a well-established *in vitro* model extensively employed for assessing numerous substances that induce changes in neuronal metabolism (rotenone, 6-OHDA, MPTP, paraquat) (Ioghen et al., 2023). It serves as a valuable model for investigating molecular processes associated with various pathologies, notably PD. Nevertheless, the considerable number of contradictory results and wide range of dopaminergic markers expression makes very difficult to compare and reproduce reported results for this cell line. The variability in culture media, differentiation protocols, and supplements used has sparked significant debate about their utility, particularly due to the discrepancies in marker expression that arise between the different protocols for growth and maintenance. This highlights the crucial need for comprehensive post-differentiation catecholaminergic characterization, with particular emphasis on molecules linked to the dopaminergic phenotype, such as tyrosine hydroxylase, dopa decarboxylase, dopamine, and its recycling transporters. SH-SY5Y does acquire neuronal phenotypes with different differentiation compounds and protocols, such as retinoic acid (RA), staurosporine (Stau), phorbol esters, brain-derived neurotrophic factor (BDNF), among others (Carvajal-Oliveros et al., 2022; Filograna et al., 2015). Nevertheless, the diversity of substances that can differentiate these cells from a neuroblast-like state into mature human neurons also represents one of the significant disadvantages of using this *in vitro* model. There is a vast array of existing and published protocols for neuronal differentiation, with variations ranging from the concentrations of the different substances used for differentiation, as well as the culture medium employed, treatment times, and concluding with the characterization and verification of the phenotypes acquired after treatment (Xicoy et al., 2017).

Recent reports have compared various aspects of the SH-SY5Y cells with other neuroblastoma cell lines that show significant promise for neurodegenerative disease research. One of the main characteristics of BE (2)-M17 is that it displays a robust dopaminergic phenotype even without a differentiation treatment making this cell line an ideal candidate for use as a research model to study the biology of dopaminergic neurons and PD (Carvajal-Oliveros et al., 2022; Filograna et al., 2015). When selecting the most appropriate catecholaminergic cell line, it is important to consider the following key characteristics of dopaminergic neurons: the capacity to synthesize dopamine, expression of VMAT-2, formation of neuromelanin, and presence of the dopamine transporter (DAT) and monoamine oxidase (MAO). In comparison to SH-SY5Y, these attributes are inherently more pronounced in BE (2)-M17.

## 3 Introducing the newcomer: the BE (2)-M17 cell line

**FIGURE 1:**
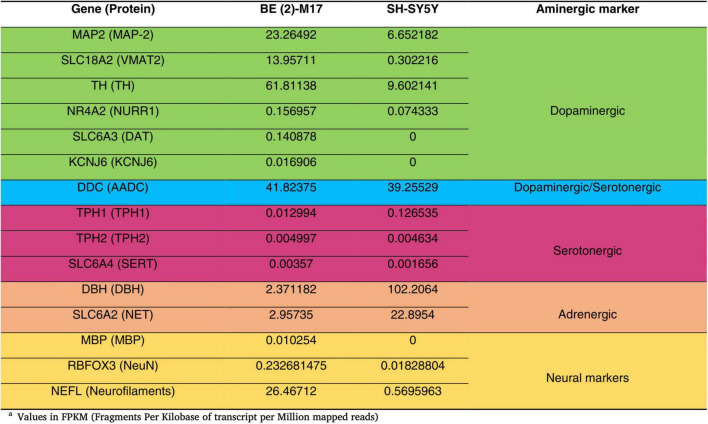
Comparison of the gene expression levels of catecholaminergic markers in undifferentiated BE (2)-M17 and SH-SY5Y cell lines. BE(2)-M17 cells exhibit higher expression of genes associated with dopaminergic (green) and neuronal (yellow) phenotypes compared to SH-SY5Y cells. These data were obtained from RNAseq analysis under undifferentiated conditions and values are express in Fragments Per Kilobase of transcript per Million mapped reads. Adapted from Carvajal-Oliveros et al. (2022), doi:10.1016/j.ibneur.2022.11.00.

BE (2)-M17 is a human neuroblastoma cell line derived from SK-N-BE (2), originally isolated from the brain tissue of a 2-year-old male in 1989 (ATCC, CRL-2267). This cell line expresses genes related to dopaminergic, adrenergic, and serotonergic pathways as well as neuronal markers (Andres et al., 2013; Filograna et al., 2015). Undifferentiated BE (2)-M17 cells exhibit limited neuronal projections and express markers characteristic of immature neurons such as Beta III tubulin and consists exclusively of adherent cells (N type), akin to their parental cell line SK-N-BE (2). While differentiated BE (2)-M17 cells extend long and branching processes and display hallmark markers of dopaminergic neurons (TH, SLC18A2, MAP2, NR4A2, and KCNJ6) and mature neurons (MBP, RBFOX3, and NEFL) (Filograna et al., 2015; Walton et al., 2004). Furthermore, undifferentiated BE (2)-M17 cells exhibit lower expression of markers associated with serotonergic neurons (TPH and serotonin transporter), and adrenergic markers (DBH and NET) in comparison to SH-SY5Y cells (Figure 1; Carvajal-Oliveros et al., 2022).

**FIGURE 2:**
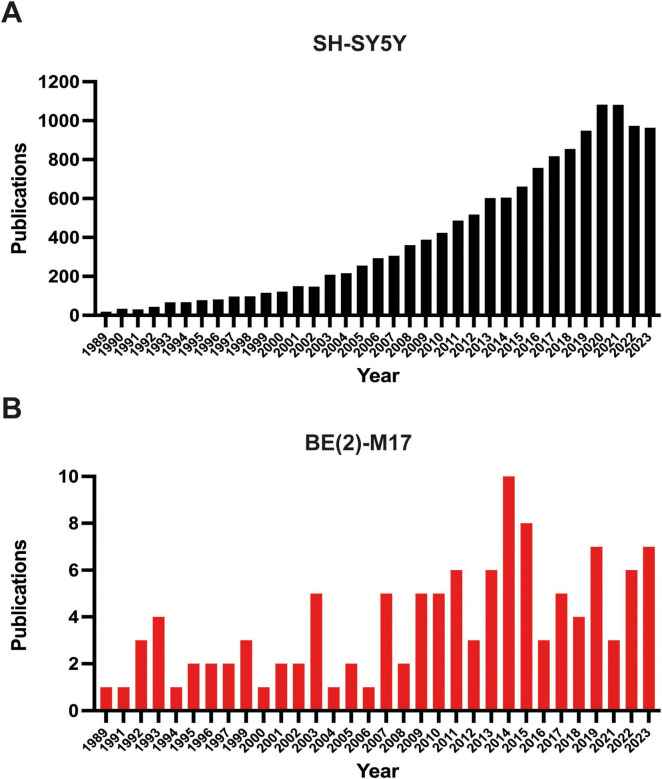
Number of publications in PUBMED (NIH). **(A)** Publications retrieved from the search “SH-SY5Y” and **(B)** from the search “BE (2)-M17.” The search was filtered starting from 1989, the year the BE (2)-M17 cell line was first reported. The graphs display the number of articles published up to the end of 2023.

**FIGURE 3:**
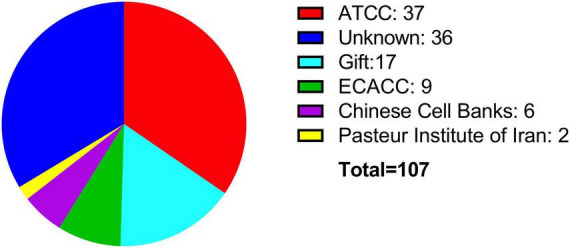
Sources of acquisition for the BE (2)-M17 cell line. Most of the cell lines have been obtained directly from commercial sources such as ATCC (red) or ECACC (green). Notably, a significant portion of publications do not specify the source of the cell line (blue).

Since its isolation, the BE (2)-M17 cell line has been used in several research fields. However, a search in PubMed with the keywords “BE (2)-M17” or “SH-SY5Y” displays 108 papers (107 articles, 1 retracted) for the BE (2)-M17 line and 12,579 results retrieved for the SH-SY5Y cell line (Figure 2) (NIH, PubMed^®^, May 2024). This difference in the number of publications between both neuroblastoma cells highlight the insufficient characterization of the BE (2)-M17 cell line. In contrast SH-SY5Y cell line has undergone exhaustive characterization and has emerged as an *in vitro* model not only for studying neurodegenerative diseases but also for a range of other research areas. While the BE (2)-M17 is commercially available, a review of the 107 publications retrieved from the PubMed search reveals that only 50.5% of the studies declared obtaining the cells from a formal cell bank (Figure 3). The lack of information regarding the origin of the cell lines used in research, along with the lack of verification of markers associated with the original cellular phenotype, has the potential of reducing reproducibility between groups and laboratories caused by selection of derivative or divergent lines with divergent phenotypes and genotypes; and there is also the risk of cell line contamination with other cells lines, human or from different animal species; as it has already been documented in the SH-SY5Y cell line, with some instances traced back to institutions where the cell lines have been maintained for over 5 years (Jiang and Wang, 2014).

**FIGURE 4:**
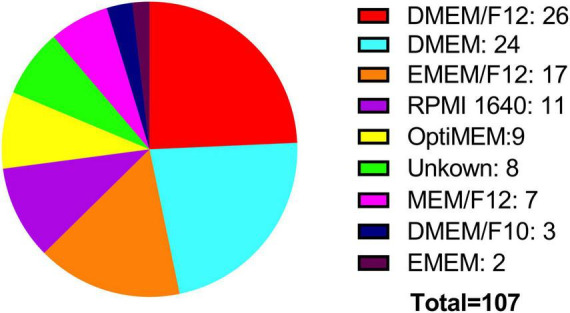
Medium used for cell culture. Two of the main cell banks recommend using EMEM/F12 (orange), while most publications report using high-glucose media such as DMEM/F12 (red). Notably, there is significant variation in the types of media used for culturing this cell line.

**FIGURE 5:**
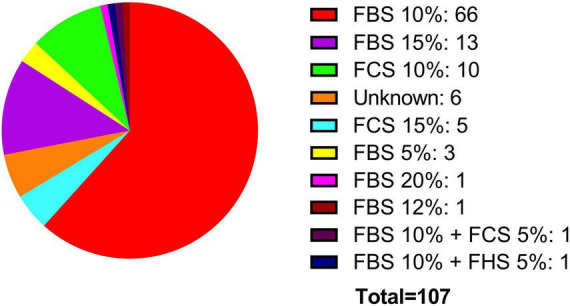
Supplementation with fetal serum. FBS has been predominantly used, typically at a concentration of 10% (red). Additionally, other types of fetal serum, such as horse (blue) and calf (cyan), have also been utilized.

Two crucial parameters to be considered in *in vitro* experiments with cell lines are the growth medium and the percentage of FBS. Both the ATCC and the ECACC, recommend in their technical data sheets for the BE (2)-M17 cell line, to use a growth medium consisting of a 1:1 mixture of Eagle’s Minimum Essential Medium and Ham’s F12, with medium renewal recommended every 4–7 days. Despite this recommendation, most publications indicate the use of DMEM and Ham’s F12, with some publications also utilizing other media such as MEM, OptiMEM, or RPMI 1640 to a lesser extent (Figure 4). In other cellular contexts, there is compelling evidence that opting for one medium over another might potentially influence cell proliferation, viability, differentiation, gene expression, and nuclear volume and morphology. This effect primarily arises from variations in glucose concentrations, with subsequent consideration of other compounds such as calcium and phosphate (Mukherjee and Weinstein, 1986; Wu et al., 2009). An additional variable in culture conditions is the FBS concentration that is used in the cell culture medium. The ATCC recommends using FBS at a concentration of 10%, whereas the ECACC suggests a concentration of 15% (ATCC, CRL-2267, ECACC, 95011816). The analysis of publications related to the BE (2)-M17 cell line indicates that FBS has predominantly been used, mostly at a concentration of 10%, with some variations between 5 and 20%. The implications for the physiology of BE (2)-M17 cells of serum concentration should be addressed and standardized in future studies, especially if culture conditions impact the neural phenotype and differentiation potential (Figure 5).

Like SH-SY5Y differentiation protocols, there is not a unique differentiation protocol to increase neuronal phenotype. The supplementation with retinoic acid (RA) has been the main method for inducing differentiation toward a neuronal phenotype in the BE (2)-M17 cell line. However, other methods have also been employed, including Staurosporine, TPA, and various compounds added alongside RA, such as BDNF. A drawback of the use of BE (2)-M17 cell line, as with other cell models, is the considerable variability of culture and differentiation conditions (Table 1). Although RA is used in most differentiation treatments, the concentrations vary from 1 to 20 μM. Additionally, the duration of differentiation is notably diverse, ranging from 1 to 12 days, along with the use of various culture media. Evidence indicates that the number of differentiation days can influence the expression of dopaminergic markers such as TH, even when using the same molecule (Carvajal-Oliveros et al., 2022; Filograna et al., 2015).

**TABLE 1:** Protocols used in the BE (2)-M17 cell line to promote neuronal differentiation.

Time and agents for differentiation	Medium	Phenotype or neuronal differentiation marker	References
5 μM RA 3–5 days	MEM and Hams F-12 FCS 15%	Growth arrest	Stephanou et al., 1992
5 μM RA 5 days	MEM and Hams F-12 FCS 15%	Growth inhibition and decrease in the apoptotic index related to neuronal differentiation	Piacentini et al., 1992
RA	Data not reported	Endopeptidase activity related to neuronal differentiation	Carvalho et al., 1993
5 μM RA 2–8 days	MEM and Hams F-12 FBS 15%	Reference to slight increase in neurite outgrowth and minor cell growth inhibition	Carvalho et al., 1993
5 μM RA 1–5 days	Data not reported FBS 15%	Slight increase in neurite outgrowth	Melino et al., 1993
5 μM RA 5 days	DMEM/F12 FBS 15%	Corticotropin-releasing factor mRNA expression	Kasckow et al., 1994
5 μM RA 3 días	DMEM/F12 FBS 15%	Synthesize and secrete corticotropin-releasing factor	Kasckow et al., 1995
1 μM RA or RA analogs 1–5 days	RPMI 1640 FBS 10%	Not neuronal markers reported after differentiation	Draoui et al., 1997
10 μM 3 days	DMEM/HAMSF12 FBS 10%	Expression of neuronal genes (Arginine Vasopressin and corticotropin releasing hormone)	Yoshida et al., 2006
20 μM RA-50 ng/ml BDNF 3–5 days	Data not reported	Neurite outgrowth	Zhang et al., 2010
1–10 μM RA 2–3 días	EMEM/F12 FBS 10%	Synapsin-1/2 and tubulin β3 expression, neuritic outgrowth, NSE, SNAP-25, synapsin, neurofilaments M and H, nAChR, mAChR, and ChAT. Increased glycine release, increase in ^45^Ca2+ uptake due to increasing KCl concentrations.	Andres et al., 2013
10 μM RA 4 days	EMEM FBS 10%	Decreased proliferation, increased number of neuritic processes, and increased length of neuritic processes.	Macias et al., 2014
10 μM RA 4 divisions	DMEM/F12 FBS 10%	Expression of key components of the amyloidogenic pathway	Leirós et al., 2015)
5 μM RA 3 days	DMEM/F12 FBS 15%	No neuronal markers reported after differentiation	(Jurek et al., 2015)
10 μM RA 4 divisions	DMEM/F12 FBS 10%	Expression of key components of the amyloidogenic pathway	(Leirós et al., 2015
30 nM TPA, 5 μM RA, Stau 8 nM 4 or 7 días	DMEM/F12 FBS 10%	Growth inhibition, neuritic outgrowth, tubulin β3 and neurofilament expression, dopaminergic, adrenergic and glutamatergic markers, dopamine and noradrenalin detection	Filograna et al., 2015
10 μM 5 days	DMEM FBS 10%	Unable to differentiate cells	Delic et al., 2017
10 μM RA 4 days	DMEM/F12 FBS 10%	Tyrosyne hydroxylase, GDNF and voltage-dependent L-type Ca^2+^ channel, α-1C subunit (CACNA1C) expression	Tavakol et al., 2019
10 μM RA/ Stau 10 nM 6 or 12 días	DMEM/F12 FBS 10%	Growth inhibition, neuritic outgrowth, dopaminergic and serotoninergic markers expression	Carvajal-Oliveros et al., 2022
1 μM RA 2 days	DMEM/F12 FBS 20%	Increased stemness and tubulin β3 expression	Sohrabi et al., 2023
5 μM RA 1 day	DMEM/F12 FBS 10%	Neurite outgrowths	Yang et al., 2023

**FIGURE 6:**
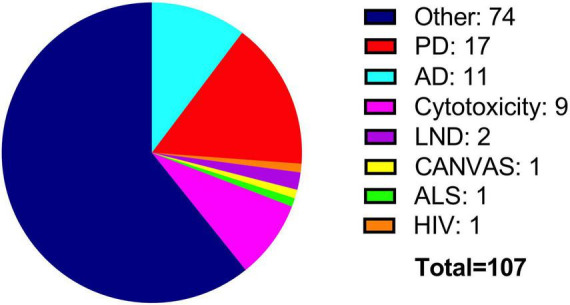
Use of the BE (2)-M17 cell line in research. Most studies have focused on the characterization properties of the cell line (blue). Additionally, BE (2)-M17 cells have been used to investigate main neurodegenerative diseases such as PD (red) and AD (cyan).

It is crucial to highlight that the substance used for differentiation leads to significant variations in the expression of certain markers. Staurosporine, for instance, significantly elevates the levels of TH and VMAT2. In contrast, RA treatment increases these markers compared to the undifferentiated state, but to a lesser extent than staurosporine. However, DAT expression is more enriched when differentiated with RA. Thus, standardizing a universal protocol is essential for the reproducibility of experiments across different contexts (Carvajal-Oliveros et al., 2022). In our experience, 6 days of differentiation with RA is an ideal time of treatment to assure maximum expression of dopaminergic markers without compromising cell viability. The BE (2)-M17 cell line has been employed in research of different pathologies (Figure 6). As the BE (2)-M17 cell line originated from a human neuroblastoma, it has served as a model for characterizing diverse effects associated with the onset and progression of tumors. Moreover, it serves as a model for evaluating drugs and their mechanisms of action utilized in the cancer treatment. In this context, researchers have assessed on the BE (2)-M17 cell line, the impact of Carfilzomib, a drug used to treat adults with multiple myeloma (Lee et al., 2019). These tumors, being the most prevalent extracranial neoplasm in childhood, contribute substantially to mortality rates among children (Nakagawara et al., 2018). The BE (2)-M17 cell line emerged as a valuable model for assessing the impact of Carfilzomib. There is a decrease in cellular viability through induction of cell cycle arrest at the G2/M phase. Furthermore, there was an observed upregulation in the activation of various proteins linked to cellular death, including caspase 8, caspase 9, and caspase 3, accompanied by other alterations such as morphological changes, an increase in endoplasmic reticulum stress and reactive oxygen species, and mitochondrial dysfunction (Lee et al., 2019). These findings demonstrated Carfilzomib’s efficacy in treating various types of cancers, particularly those that impact young people.

### 3.1 Exploring new avenues: the BE (2)-M17 cell line as a model for Parkinson’s disease

Recently, the BE (2)-M17 cell line has been established as a solid dopaminergic model according to *in vitro* assays in human systems. These cells have been used to study the effects of various drugs that primarily target mechanisms associated with PD. The toxicity models using BE (2)-M17 cells employ neurotoxins such as 6-hydroxydopamine (6-OHDA) and rotenone to replicate pathological characteristics observed in PD. The mechanism of action of 6-OHDA involves its internalization through dopamine (DAT) and norepinephrine (NAT) receptors, leading to increased oxidative stress levels in the cells (Simola et al., 2007). Rotenone is known to primarily disrupt the mitochondrial respiratory chain, leading directly to an increase in oxidative stress (Heinz et al., 2017). The exposure of BE (2)-M17 cells to these toxins induces oxidative stress, mitochondrial dysfunction, and neuronal death, thus providing a valuable model for understanding the toxicity mechanisms associated with PD (Xiong et al., 2012).

The BE (2)-M17 cell line has also been used for the evaluation of the processes related to PD and has led to the identification of potential therapeutic targets. For example, the G-substrate can activate mechanisms that reduce the vulnerability of this cells to 6-OHDA-induced damage. Although this cellular mechanism is not fully understood, the G substrate has been associated with increased levels of pAkt, which is linked to the inhibition of serine/threonine protein phosphatase activities closely related to apoptosis and DNA damage responses. Proteins like protein phosphatase 2 (PP2A) and their inhibitory functions have been closely associated with reducing the vulnerability of neurons in PD models (Chee et al., 2007). Additionally, the effects of MG-132, a reversible inhibitor of the 26S proteasome, have also been examined. This inhibitor disrupts the proper degradation of ubiquitinated proteins, contributing to the accumulation of misfolded proteins, which is relevant for studying the pathological mechanisms of PD (Chee et al., 2007).

An advantage of the BE (2)-M17 cell line is that its neuronal phenotype allows the study of the molecular machinery that synthesizes and subsequently release neurotransmitters. This includes the expression of neuron-associated proteins, such as neurotransmitter recycling transporters and specific receptors (Carvajal-Oliveros et al., 2022; Filograna et al., 2015). Additionally, the synthesis of enzymes responsible for the degradation of these neurotransmitters allows for a multifaceted evaluation of the disruption of normal dynamics in a neuronal environment, thereby providing a direct focus on the pathology of PD. The disruption of proper neuronal function, particularly by molecules involved in the release and degradation of neurotransmitters, has been identified as a significant factor in the development of PD pathology. VMAT2, a transporter crucial for the synaptic vesicular packaging of monoamines such as serotonin, norepinephrine, histamine, and dopamine, has emerged as an important target for PD treatment (Eiden and Weihe, 2011). Low levels of VMAT2 are associated with an increased risk of developing the disease, and postmortem samples from PD patients show a notable reduction in VMAT2 levels. Furthermore, various models have demonstrated that alterations of VMAT2 levels lead to evident neuronal dysfunction. For instance, VMAT2 deficient animals experience dramatic dopamine depletion, progressive loss of dopamine neurons, and α-synuclein accumulation (Bridi and Hirth, 2018; Taylor et al., 2011). Conversely, mice with elevated VMAT2 levels exhibit an increased capacity for dopamine storage in their neurons, resulting in higher total dopamine levels, increased dopamine release, and protection from neurotoxic insult by MPTP (Lohr et al., 2016). The BE (2)-M17 cells, exhibit high basal expression of VMAT2, have been employed to assess the effects of various compounds on the functionality of this transporter. Tricyclic and tetracyclic antidepressants, such as amitriptyline, desipramine, imipramine, protriptyline among others, have shown significant upregulation of VMAT2 activity. Notably, the expression levels of VMAT2 mRNA, along with other transporter mRNAs expressed by this cell line, including DAT, SERT, VMAT1, and NET, remain unchanged (Wang et al., 2024). The BE (2)-M17 cell line thus provides a valuable platform for further research into VMAT2 and its interactions, particularly with the DAT transporter. This focus is crucial for studying the alterations associated with PD and the dysfunctions affecting the proper release of dopamine.

The accumulation and aggregation of proteins, mainly α-synuclein, are hallmarks of PD (Calabresi et al., 2023). Studies using BE (2)-M17 cells have demonstrated that overexpression of α-synuclein leads to its aggregation and subsequent cellular toxicity. This has enabled researchers to explore the mechanisms through which this protein contributes to PD pathogenesis and to evaluate compounds that may inhibit its aggregation. In this context, BE (2)-M17 cells have been employed to investigate the interactions of less-studied proteins in PD that, despite their limited exploration, play crucial roles as negative regulators of cell death pathways. These proteins could serve as important focal points for understanding the disease mechanisms. Among them, 14-3-3 proteins are a conserved group of eukaryotic proteins involved in various processes, including signal transduction, cell cycle regulation, malignant transformation, stress response, and apoptosis (Fu et al., 2000). Studies have shown that α-synuclein directly interacts with one isoform of the 14-3-3 proteins, known as 14-3-3s, which plays a critical role in cellular survival. Notably, this interaction has been observed in Lewy bodies, a hallmark of PD (Kawamoto et al., 2002). Overexpression of specific isoforms, such as 14-3-3θ, -ϵ, and -γ, in BE (2)-M17 cells led to reduced toxicity from rotenone exposure and decreased accumulation of the insoluble form of α-synuclein. Similar effects were observed with MPP+, another toxin used in PD research. One of the mechanisms underlying these observed results is a diminished ability of α-synuclein to sequester 14-3-3 proteins. This reduction can lead to cell death activation, as it allows the release of apoptotic factors that are typically inhibited by 14-3-3 proteins (Yacoubian et al., 2010).

The advantage of using a model with endogenous dopamine synthesis such as BE (2)-M17 cell line, along with its regulatory and synthesis machinery, has allowed the study of additional aspects related to this important neurotransmitter. Research suggests that dopamine itself may contribute to dopaminergic neuron death due to intracellular accumulation resulting from dysfunction in presynaptic vesicle release mechanisms (Cramb et al., 2023). This accumulation initiates dopamine degradation processes and an increase in metabolites such as DOPAC and quinones, which promote increase in the levels of free radicals and oxidative stress (Segura-Aguilar and Paris, 2014). In this context, studies have focused on α-synuclein. A primary function of this protein is closely linked to- the proper assembly of the SNARE complex, which plays a key role in presynaptic vesicle release (Yoo et al., 2023). Research indicates that simply overexpressing either the wild-type (WT) α-synuclein or its A30P mutant significantly reduces cell viability of the BE (2)-M17 cells. Additionally, the overexpression of α-synuclein and its A30P mutant increases susceptibility to compounds such as dopamine and L-DOPA, decreasing viability by up to 57% for the WT protein and up to 63% for the A30P mutant (Bisaglia et al., 2010).

A role of α-synuclein in lysosomal pathways such as chaperone-mediated autophagy has been suggested in BE (2)-M17 cells exposed to MPP+ (Eriksson et al., 2017). The lysosomal function is disrupted by increased oxidative stress by promoting lysosomal membrane permeabilization which is believed to contribute to neuronal death through the release of hydrolytic proteases into the cytosol (Dehay et al., 2010). The BE (2)-M17 cell line has been instrumental in studying cholesterol dynamics within a neurodegeneration model using MPP+, a toxin widely employed to induce Parkinsonian phenotypes. MPP+ inhibits mitochondrial complex I, leading to increased oxidative stress (Li et al., 2024). MPP+ exposure increases lysosomal cholesterol levels and stimulates α-synuclein aggregation. It seems that enhanced lysosomal cholesterol levels might be a protective mechanism induced after mitochondrial disfunction which may help to prevent lysosome membrane permeabilization (Appelqvist et al., 2012; Eriksson et al., 2017). These findings show how BE (2)-M17 cells can be employed to understand additional molecular mechanisms in PD pathology, advancing our understanding of cellular organelle dynamics in dopaminergic neurodegeneration.

The dopaminergic phenotype of the BE (2)-M17 cell line has also been crucial in studying various molecules associated with neuronal death and their potential impact on mechanisms related to the progression of PD. It is well established that heavy metals are one of the environmental factors that promote the development of this pathology (Pyatha et al., 2022). The presence of Fe (II) makes the dopaminergic BE (2)-M17 cells more susceptible to DNA when α-synuclein is overexpressed, and this effect is even more toxic when the mutant A53T form of α-synuclein is overexpressed (Martin et al., 2003). BE (2)-M17 cells have also been employed to assess a range of neuroprotective compounds that may offer potential benefits in treating PD. These studies are essential for discovering new therapeutic strategies and moving toward more effective treatments for PD. In this context, cinnamic aldehyde, a key flavor compound in cinnamon essential oil, has been identified as an antioxidant and anti-inflammatory agent (Chen et al., 2022). Cinnamic aldehyde demonstrated a protective effect in a cytotoxicity model induced by MPP+. When the BE (2)-M17 cell line was exposed to 0.5 mM MPP+, cell viability was reduced by 50%. Remarkably, this reduction was reversed by treating the damaged cells with 10 μM cinnamic aldehyde for 48 h. The protective mechanisms of cinnamic aldehyde have been linked to the regulation of autophagy, primarily through the modulation of Microtubule-associated protein 1A/1B-light chain 3 (LC3), a key protein in the autophagy pathway, and sequestosome 1 (p62), which is crucial in the protein ubiquitination process (Bae et al., 2018).

## 4 Conclusion

Over the years, various models have been developed to understand the mechanisms associated with the onset, development, and progression of neurodegenerative diseases. In vitro models, specifically immortalized cell lines, continue to represent one of the best options of study models due to several key advantages. Many of these immortalized cell lines are readily available for purchase through cell banks and other suppliers. This easy access to well-characterized and validated cell models is crucial for ensuring reproducibility and enabling the comparison of results across different laboratories. The BE (2)-M17 cell model is an attractive alternative tool in PD research because it effectively simulates key aspects of the pathogenesis of this neurodegenerative disorder. Studies that used the BE (2)-M17 cell line have yielded important insights into the pathological mechanisms of PD and facilitated the evaluation of potential therapies. Nonetheless, like in all *in vitro* models, these studies have limitations and challenges. Ongoing development and refinement of these models are crucial for deepening our understanding of PD and advancing toward more effective treatments. The neuronal characteristics of the BE (2)-M17 cell line and its inherent expression of dopaminergic markers, even in the absence of differentiation protocols, provide significant advantages to extend its application to PD research and other neurodegenerative diseases. However, the full potential of BE (2)-M17 cells line as a model of PD and other dopaminergic alterations remains to be exploited. the molecular characteristics of this cell model expand its utility for the rapid and effective evaluation of various potentially beneficial compounds for dopaminergic system.

The BE (2)-M17 cell line offers significant advantages over the SH-SY5Y line. Firstly, its basal expression of TH, DAT, and VMAT2 enables a faster and more efficient study of effects on the dopaminergic synthesis pathway without requiring differentiation treatments. Additionally, the use of various drugs and toxins to replicate neurodegeneration mechanisms in this type of model is essential, as it is important to understand both the mechanisms of action of these toxins and whether the model being used expresses the receptors or molecular machinery necessary for these toxins to induce cellular damage. A clear example is the use of 6-OHDA, a dopaminergic analog that is taken up into the cell through DAT (Hernandez-Baltazar et al., 2017). Studies suggest that the SH-SY5Y cell line does not express this transporter. When comparing the concentrations needed to reduce cell viability, the SH-SY5Y line proves to be more resistant, as it only shows viability alterations at concentrations above 100 μM (Elyasi et al., 2021). In contrast, the viability of as the neuroblastoma IMR-32 cell line, which also express DAT, and BE (2)-M17 cell line is affected by concentrations around 50 μM (Czech et al., 2014; Iglesias González et al., 2019).

The characteristics of BE (2)-M17 cells outlined in this review indicate that this cell line either has similar properties or outperform the SH-SY5Y cell line in certain parameters. Thus, BE (2)-M17 can be considered as a primary model for comparison and to study particular aspects of the dopaminergic pathway in the context of PD. A broad array of cellular models is essential for studying enigmatic diseases such as PD and other neurodegenerative diseases. Despite the progress in our knowledge of PD over the last three decades, there are still several cellular mechanisms that must be studied in greater depth to understand how to mitigate its symptoms and in the best-case scenario how to halt its progression. We encourage the PD research community to include the BE (2)-M17 cell line as an extra tool in the portfolio of research resources.
